# Reasons for Seeking HIV-test: Evidence from a Private Hospital in Rural Andhra Pradesh, India

**DOI:** 10.3329/jhpn.v26i4.1885

**Published:** 2008-12

**Authors:** Sudha Sivaram, Gurcharan Singh Saluja, Manik Das, P. Sudhakar Reddy, Vijay Yeldandi

**Affiliations:** 1 Infectious Diseases Program, Department of Epidemiology, Johns Hopkins Bloomberg School of Public Health, 615 North Wolfe Street, Room E6610, Baltimore, MD 21205, USA; 2 Mediciti Immunology and Infectious Disease Research Institute, SHARE, India, Ghanpur Village, Medchal Mandal, R.R. District 501401, India; 3 School of Medicine, University of Pittsburgh, Pittsburgh, USA, F751 UPMC Presbyterian, Pittsburgh, PA 15238, USA; 4 Stritch School of Medicine, Loyola University,445 East North Water Street, Suite 701, Chicago, IL 60611, USA

**Keywords:** HIV, HIV infections, Acquired immunodeficiency syndrome, Private sector, Rural health services, Voluntary counselling and testing, India

## Abstract

This study sought to describe the development of HIV counselling and testing services in a rural private hospital and to explore the factors associated with reasons for seeking HIV testing and sexual behaviours among adults seeking testing in the rural hospital. Data for this study were drawn from a voluntary counselling and testing clinic in a private hospital in rural Andhra Pradesh state in southern India. In total, 5,601 rural residents sought HIV counselling and testing and took part in a behavioural risk-assessment survey during October 2003–June 2005. The prevalence of HIV was 1.1%. Among the two reported reasons for test-seeking—based on past sexual behaviour and based on being sick at the time of testing—men, individuals reporting risk behaviours, such as those having multiple pre- and postmarital sexual partners, individuals whose recent partner was a sex worker, and those who reported using alcohol before sex, were more likely to seek testing based on their past sexual behaviour. Men also were more likely to seek testing because they were sick. The findings from this large sample in rural India suggest that providing HIV-prevention and care services as part of an ongoing system of healthcare-delivery may benefit rural residents who otherwise may not have access to these services. The implications of involving the private sector in HIV-related service-delivery and in conducting research in rural areas are discussed. It is argued that services that are gaining prominence in urban areas, such as addressing male heterosexual behaviours and assessing the role of alcohol-use, are equally relevant areas of intervention in rural India.

## INTRODUCTION

An important area of focus for HIV-prevention and care services in India is effective outreach and service provision to its rural population. Over 75% of over one billion people of India live in rural areas (1). In a national HIV surveillance, the prevalence of HIV among women attending rural antenatal clinics was 0.57% compared to 0.64% in urban clinics; however, the prevalence of HIV was higher in some southern states than in rural areas. Rural residents have lesser information relating to HIV than their urban counterparts (2) and have poorer availability and access to general health services (3). Further, the quality of HIV services in rural areas is often characterized by unqualified physicians (4,5), medical practitioners refusing to care for HIV/AIDS, and poor adherence to confidentiality and other HIV-testing principles that safeguard rights of individuals and nurture trust with providers (6).

To develop the needed high-quality services for the prevention and care of HIV for rural residents in India, it is important to learn from approaches that have been successful elsewhere. Results of a review of literature suggest two possible approaches—the first one is the role of voluntary counselling and testing (VCT) centres (7,8), and the second one is the increasing role of health service-delivery led by the private sector in rural areas (9-11). By offering accurate information about risks of HIV and realistic options about living with HIV if an individual tests positive, VCT services help individuals specifically prepare for modifications in sexual and treatment-seeking behaviours (12). Results of studies in urban India among VCT clinic attendees have demonstrated that VCT attendance was associated with lower risk behaviours, increased knowledge, and improved ability to seek follow-up testing and care (13,14). A feature common to these largely stand-alone clinics may be their ability to offer high-quality and non-judgmental services, maintain confidentiality, and enable follow-up care (15). While there is a consensus that such services are lacking in India, the literature suggests that these can, indeed, be developed in resource-poor settings. Large public-health initiatives in India (9,16) and elsewhere (17), while working towards different health outcomes, have a common approach. They are private-sector initiatives, with committed and sustained leadership and highly-trained staff who work within the demands and constraints in the local community.

An important feature of rural health services worldwide is the ability to offer several services at one location (18). This model of integrating services enables care-seekers to consult with a range of providers in a single location; it also enhances provision of care, monitoring and evaluation of various services (19). This potential of the private sector for offering HIV services remains to be investigated in rural India. Current evidence further bolsters the case for such an investigation. Over 89% of all health practitioners in India work in the private sector (3). Individuals prefer seeking HIV/STD-related care from the private sector (20). However, a large proportion of care providers adheres poorly to the standards of patient interaction and counselling, has substandard ethical principles, and has a wide range of practices relating to clinical management of HIV/STDs (21). Further, there is also a lack of outreach efforts to inform and educate rural residents about HIV in India (22). This resulting combination of uninformed consumer and unqualified care providers is a significant barrier to care and prevention services in rural India.

In this study, we have a unique opportunity to collaborate with a rural private hospital based in the southern Indian state of Andhra Pradesh to develop HIV-prevention and VCT services. The Government of India categorizes Andhra Pradesh as a ‘high-prevalence’ state based on a prevalence of HIV over 5% in high-risk groups, such as sex workers and over 1% in traditionally low-risk groups, such as pregnant women (23). Further, a recent analysis of rural government-run VCT clinic populations in Andhra Pradesh concluded that the prevalence of HIV from these clinics may over-represent actual prevalence because of an inherent selection bias in the client population (24). Clients are relatively poor, at a higher risk for HIV largely due to referrals from the private sector, and underserved (24). To develop prevention and care services for these individuals, we need to understand motivations for test-seeking and identify risky sexual behaviours among rural residents. This understanding might add to available approaches and strategies to serve this population.

In this study, we had two aims. First, we sought to describe the development of HIV counselling and testing services in a rural private hospital. Second, we sought to explore the factors associated with reasons for seeking HIV testing and sexual behavi-ours among adults seeking testing in the rural hospital. We note here and describe in the next sectionthat this data collection was part of a larger project where we established a VCT clinic in a private hospital and conducted prevention-education programmes for the residents following which we provided VCT services.

## MATERIALS AND METHODS

### Setting

The study was implemented in a rural block of 40 villages located in southern part of the Andhra Pradesh state in southern India. The villages, apart from government-run health centres, were also served by the Mediciti Hospitals, a private hospital and referral centre to other public and private clini-cs in the area. This hospital is located about 40 km from Hyderabad city, the state capital. The infectious disease programme at Mediciti is spearheaded by the Mediciti Immunology and Infectious Disease Research Institute (MIIDRI). The activities reported in this paper have been implemented in collaboration with the MIIDRI.

### Existing community outreach efforts

The community outreach programme at the Mediciti Hospitals, called REACH (Rural Effective and Comprehensive Health), was established in 1996. Through the mandate of REACH, Mediciti trains community health volunteers (CHVs) to monitor the health in the community. The REACH programmes provide comprehensive primary care education and outreach services to residents in all 40 villages. These services include immunization, growth monitoring, and maternal and child nutrition. The CHVs periodically collect all details of population and status of health, and a secure computerized database is maintained. In this database, every household and individual within the household has an ID number. The physicians and hospital administration use this number to monitor service-delivery and follow-up care. This database helped ascertain the sampling frame of this study and plan for outreach for HIV education in this population. To provide easy access to the hospital, buses operate between key locations in the area to the hospital.

### Development of VCT programme

This involved (a) identification of the target population, (b) development of logistics of VCT provision, (c) provision of HIV-prevention education, and (d) provision of VCT services.

*Identification of target population*: Adults aged 18-55 years and population at risk consistent with other studies in India and elsewhere were chosen in our study to be eligible for VCT services (25). Information on size, demographics, and location of these individuals was obtained from the REACH database. There were 30,000 adults aged 18-55 years in a population of 68,523 residents in the 40 study villages. The VCT programme sought to serve these eligible adults.

*Logistics of VCT provision*: The REACH programme assigned a CHV to each of the 40 villages. The CHV was responsible for monitoring health of the residents of their assigned village(s). Monitoring included several activities, such as providing reminders for follow-up appointments and immunizations, recording birth/death and migration information, and providing health education and referrals. Residents visit the hospital at least once in three months for routine health education and follow-up. The hospital provides free transportation from all the villages to the hospital on a daily basis. Given the existing access to the hospital, VCT services were housed in the hospital in the MIIDRI wing in a corridor on the rear side of the building. There was no sign or notification informing the availability of HIV testing. This was done to minimize stigma. Private cubicles for counselling were erected, and the procedures for registering patients were developed. These procedures involved the development of password-protected databases of resident information so that information on HIV-testing and results can be maintained confidentially and separately from the main REACH database. Participants arriving at the hospital were informed prior to and at the time of their visit for available VCT services.

*Provision of HIV-prevention education*: To inform individuals about VCT services prior to hospital visit, we conducted education activities in all the 40 villages. Most programmes were village-based and included some or all the following elements: interactive audiovideo presentation, group discussions, and HIV information sessions in various village forums. In addition, implementation staff, along with REACH staff, visited households, offered information on HIV prevention, and answered questions that residents had raised. Integral to education efforts was provision of information on HIV counselling and testing and the availability of these services at the Mediciti Hospitals.

*Provision of VCT services*: With funding available for this study, the MIIDRI hired five counsellors and four field staff members for the study. All hired staff and hospital management first underwent training in ethical principles in general and as they apply for HIV-related education and care services. Further, the field staff members were trained in research methods, such as ethnography and survey, and community outreach for HIV prevention and care. The field staff and counsellors were assigned villages to focus on, and before the activities began, they met the CHVs in their respective villages. Given their rapport with the community, the CHVs were consulted on various aspects of the study.

The CHVs and field staff informed the residents about the availability of VCT services for HIV at the hospital. Several individuals informed us that services would be more accessible if these are available at the community level. Depending on the convenience of the individuals, they could choose to visit the VCT centre at hospital or at an MIIDRI field office in the community. While they could visit the hospital any day as part of their routine health consultation, arrangements were made at the MIIDRI field office to offer these services on a weekly basis. Transportation was not provided to these field offices. The field offices also offered health education and were manned by health professionals, including a nurse and doctors.

Visit of the individuals to the VCT centre was scheduled after their planned hospital consultation. In the hospital complex, the staff directed the participants to the location of various services of interest to them.

Once participants arrived for VCT, regardless of venue, they registered at the clinic. Registration required providing information about the hospital ID number of the patient to facilitate future contact for post-test counselling and any needed follow-up. Participants were then administered to provide informed consent to take part in a risk-assessment survey and VCT. Some individuals chose to reschedule their visit. For these individuals, the counsellors scheduled and facilitated an appointment at another convenient time. Those who were willing proceeded for pretest counselling and testing after the survey. Trained counsellors filled out the questionnaire on paper. The survey asked questions on sexual behaviour and substance-use behaviour to assess risk of exposure to HIV and to understand motivators for volunteering for an HIV-test. Blood samples were collected from the participants and tested twice by enzyme-linked immunosorbent assay (ELISA) (GenScreen Bio-RAD). Those samples testing positive by either one or both test(s) were confirmed using Western Blot (France Biorad LAV Blot). Participants were asked to return for the test-results in a week from the time sample was collected. Initial batches of test-results saw less than 1% of clients returning voluntarily. To inform clients of their results in a timely manner—rather than to provide reminders—the counsellors visited the villages, met the individuals, and discussed the test-results at a place of their choice. While we collected information from the counsellors on reasons for not returning for test, this was not systematically collected from the clients. The education and VCT programme reported in this paper was implemented during October 2003–June 2005.

Before beginning education activities, we also conducted ethnographic in-depth interviews (n=20; 10 men and 10 women) with a purposively-selected sample of community residents. These interviews sought to understand, from a qualitative perspective, the attitudes and beliefs of residents towards the availability of VCT services. We also elicited information on knowledge and attitudes of the res-pondents towards HIV/AIDS in these interviews. Before any personal or biological data were collected, the participants provided written informed consent. The institutional review boards of Johns Hopkins Bloomberg School of Public Health and Mediciti Hospitals approved all consent and other study procedures.

### Study variables and statistical analyses

The risk-assessment questionnaire was developed based on earlier studies in India (25) and on ethnographic data collected from the study site. We assessed two sets of behaviours in the questionnaire. First, we looked at reasons for seeking the HIV-test during the hospital visit, and second, we assessed sexual risk behaviours. The following three reasons for seeking an HIV-test were explored: past sexual behaviour, past sexual behaviour of partner, or to reconfirm the result of an earlier HIV-test. The participants were also asked for other reasons. We defined sexual risk by six variables: status of HIV, premarital sex partner, postmarital sex partner, relationship with most recent partner, history of condom-use, and alcohol-use before sex. The questions on reasons for care-seeking were assessed as a single response to one question on reasons for care-seeking; however, the participants could also cite reasons other than the three that were asked. The questions on sexual risk were assessed as categorical (the number of sexual partners coded either as none/one or at least two) or binomial responses [relationship with most recent partner was assessed as either spouse or sex worker/other casual partners; condom-use with most recent partner assessed as ever or never; history of exchanging sex for money (yes vs no)], and ever-use of alcohol before sex (yes vs no). Survey data were analyzed using the Stata software (Intercooled Stata version 8.0). First, we examined the descriptive statistics. To determine the gender differences in sexual and test-seeking behaviours, data were stratified by gender. Chi-square tests were conducted to understand the differences in reported frequencies of sexual behaviour and reasons for test-seeking. Following this, bivariate logistic regression analysis was conducted to assess the associations between various covariates and test-seeking behaviours. Finally, for those significant variables in bivariate analysis, Mantel-Haenszel common odds ratio estimates were computed adjusted for age, marital status, and history of condom-use. Ethnographic interviews were tape-recorded in the local language, translated, and transcribed in English. Transcripts were analyzed using Atlas.ti, a textual analysis software program (26). The data were reviewed for two main themes or codes as they were called in the software program: sexual behaviour and perceptions/motivations for testing. Text that matched these codes was retrieved and reviewed. Matrices were developed for each code to enable organization of the data and to understand the similarities and contrasts across related themes.

## RESULTS

We present results from ethnography and the survey. In total, 5,601 (18%) rural residents of 30,000 eligible adults visited the VCT clinic and participated in the survey. Twenty-three percent of these individuals participated at the hospital, and the remainder sought testing at the field office. All those who attended the clinic consented to take part in the survey and get tested. Of these, 1.1% were HIV-positive.

Table 1 presents the sociodemographic characteristics of the study population (n=5,601). Over half of the volunteers were aged less than 45 years, and 52% were females. Of the sample, 89% were currently married. Most (91%) earned less than 30 dollar a month, and 64% had no formal education.

**Table 1.** Sociodemographic characteristics of participants (n=5,601)

Variable	Frequency (%)
Age (years)	
18-25	1,249 (22)
26-45	1,737 (31)
45+	2,615 (47)
Marital status	
Currently married	5,008 (89)
Currently unmarried	593 (11)
Monthly income (US$)	
Less than 10	1,010 (18)
10-20	1,591 (28)
20-30	2,550 (45)
More than 30	450 (9)
Education	
No schooling	3,572 (64)
Some primary schooling	699 (12)
At least high-school education	1,085 (19)
College or higher education	245 (15)

### Reported sexual behaviours

Table 2 shows the frequency distribution of reported sexual behaviours among the participants. Nine percent of the respondents reported two or more partners before marriage, and 3% reported the same number after marriage. Two percent of the sample reported that their most recent partner was a sex worker or other casual partners. A history of condom-use was reported by 0.5% of the sample.

**Table 2.** Frequency distribution of reasons for test-seeking and sexual behaviours among individuals who sought voluntary counselling and testing provided by a rural private hospital in Andhra Pradesh, India

Variable	Frequency (%)
HIV-positive status	61 (1.1)
Number of partners before marriage (n=5,601)	
0 partner or 1 partner	5,315 (91)
2+ partners	286 (9)
Number of partners after marriage (n=5,008)	
0 partner or 1 partner	4,880 (97)
2+ partners	128 (3)
Relation with most recent partner (n=4,313)	
Spouse	4,224 (98)
Sex worker or other casual partners	89 (2)
Condom-use with recent partner (n=4,287)	
Ever	21 (<1)
Never	4,266 (99)
Alcohol-use before sex (n=5,042)	
No	4,521 (90)
Yes	521 (10)
Seeking test based on past sexual behaviour	148
Seeking test based on past sexual behaviour of partner	13
Seeking test to reconfirm a previous HIV-test	54
Seeking test because they felt sick	812
Seeking test because they were pregnant	54
Seeking test because partner was sick	5

### Reasons for test-seeking

Of those individuals who were tested, 1,086 (19%) gave a reason for attending the clinic. Most (81%) stated that they had heard about HIV and wanted to avail of testing. Table 2 presents the frequency distribution of reasons for test-seeking. Of the three reasons for test-seeking that were assessed, 3% of the participants reported seeking test based on their past sexual behaviour, and less than 1% reported seeking test based on the partner's sexual behaviour or to reconfirm a previous HIV-test result. Other reasons were also reported. Fifteen percent (n=812) of the individuals reported seeking test because they were sick, and less than 1% of the participants cited reasons of being pregnant or because their partner was sick. Of unmarried individuals, one reported seeking test as he planned to get married.

We examined the proportion of individuals who were positive for HIV against the reason given for testing. Of those who reported test-seeking because they were pregnant, 1.8% were HIV-positive. Of those who reported feeling sick as a reason to be tested, 0.6% were HIV-positive. None of those who were seeking test to reconfirm a previous test or because of the partner's sexual behaviour was positive. Of those who sought test based on their own sexual behaviours, 3.4% were HIV-positive. Of women who sought test because they were pregnant, 20% were HIV-positive.

We also examined the proportions of individuals reporting risk behaviours against reasons for test-seeking. We tallied the three risk behaviours assessed (premarital relationships, postmarital relationships, and relationship with recent partner) with various reasons for test-seeking. Of those who reported seeking test because they were pregnant, there were no reports of risk behaviours. Of those who sought test because they were at risk, 5% reported at least two partners before marriage, 2.4% reported at least two partners after marriage, and 2.7% reported that their most recent partner was a sex worker/casual partner. Of those who sought testing to confirm a past result, premarital, extramarital and risky recent partner was reported by 11%, 7.4%, and 10% respectively. Of those who sought test based on their partner's sexual behaviour, there were no reported risk behaviours. Of those who sought test based on their own sexual behaviours, premarital, extramarital and risky recent partner was reported by 48%, 32%, and 34% respectively.

In the statistical analysis, we examined covariates associated with self-reports of past sexual behavi-our and seeking test because the individuals were sick (Table 3). Other reasons were not explored due to very low response rates. In the bivariate logistic regression analysis, men with higher income and higher education and who reported a casual sexual partner/sex worker as their most recent partner had a significantly higher odds of reporting past sexual behaviour as a reason for testing. Similar trends were seen in the data for those reporting being sick as a reason for testing. The odds of this outcome were higher among men, among married individuals, and among those with a higher income and education. In the multivariate analysis (data not shown in tables) adjusted for condom-use, men (compared to women—odds ratio [OR]=47.4, confidence interval [CI] 6.5-347.8) and those reporting casual partner/sex worker as a most recent partner (OR=41.2, CI 20.4-86.4) had a significantly higher odds of stating past sexual behaviour as a reason for test-seeking. Married men compared to unmarried individuals had a significantly lower odds of stating this reason (OR=0.11, CI 0.03-0.31). In the multivariate analysis of exploring associated with being sick as a reason for test-seeking, men (OR=2.04, CI 1.6-2.6) and those reporting casual partner/sex worker as a recent partner (OR=2.8, CI 1.47-5.47) had a significantly higher odds of reporting being sick as a reason for test-seeking. Married individuals had a lower odds of reporting this as a reason (OR=0.28, CI 0.10-0.73).

**Table 3.** Statistically significant (p<0.05) covariates in the bivariate analysis of association between reasons for test-seeking (outcome) and sociodemographic and sexual behaviour variables (covariates) among individuals who sought HIV testing provided by a private rural hospital in southern India

Covariate	Seeking test because of past sexual behaviour—odds ratio (95% CI)	Seeking test because individual felt sick—odds ratio (95% CI)
Gender	170.8 (23.8-1221.5)	0.57 (0.48-0.66)
Education		
No schooling	1.0	1.0
Some primary schooling	2.7 (1.7-4.2)	0.38 (0.28-0.50)
At least high-school education	2.5 (1.7 −3.7)
College or higher education	3.0 (1.6-5.6)	0.10 (0.04-0.23)
Monthly income (US$)		
Less than 10	1.0	1.0
10-20	8.3 (1.1-63.6)	0.79 (0.66-0.95)
20-30	43.7 (6.1-313.9)	0.19 (0.15-0.24)
More than 30	66.9 (9.1-493.5)	0.17 (0.11-0.27)
Partners before marriage		
One partner or no partner	1.0	
Two or more partners	31.9 (22.4-45.5)	
Partners after marriage		
One partner or no partner	1.0	
Two or more partners	31.8 (21.1-47.8)	
Recent partner		
Spouse	1.0	
Sex worker/casual partner	35.9 (21.4 −60.2)	
Using alcohol before sex		
No	1.0	
Yes	14.9 (10.6-21.0)	

CI=Confidence interval

### Ethnographic analyses

Ethnographic data on sexual behaviour indicated some barriers to behaviour change. The respondents confirmed multiple partners among men and acknowledged stigma to purchase condoms. One male participant reported, “Young men are getting married late these days, and they are seeking jobs elsewhere and becoming more aware of their world. Also, families are arranging marriages later for men. As a result, they seek out sexual partners before marria*ge*.” Another man noted the stigma towards buying condoms: “People feel shame or guilt to buy condoms. They think that others will make a bad judgment about their sexual behaviour.”

Ethnographic data on the perceptions of the testing process, motivations, and behaviours relating to testing illustrated the role of HIV care in private practice and stigma associated with testing. More than one participant reported hearing of or seeing advertisements by Dr. X who reported having a cure for AIDS. Other private-sector clinics reportedly offered testing but not counselling. The participants felt that those being tested, and if needed, their immediate families should be informed about the test-results. Further, they also felt that the doctor should discuss with them the reasons why an HIV-test was being ordered. The participants shared that testing practices were not uniform in the area. Some men perceived that their results would not be kept confidential and that doctors would likely treat someone asking for an HIV-test with disrespect. One participant shared, “People feel that if we go to VCT in our area, they may be mistreated. Somehow others in the area will come to know. So, many feel that going to a far away place for testing is better as nobody will know.” The stigma associa-ted with HIV was a significant barrier to care-seeking. As one woman reported, “When a person has HIV, s/he will be branded as a ‘bad’ person who was either a sex worker or who has sex with a sex worker.” Another woman remarked, “People will avoid talking to them; family members will restrict their movement; and friends and other relatives will not visit them. As a result, they may not reveal need for testing or even an STD to family members. They will go far away to get treated.”

## DISCUSSION

The study individuals who sought an HIV-test were predominantly married, had a little-to-no education, and earned a very little income. The study helps understand three aspects of HIV service-delivery as these pertain to the rural Indian context: development of VCT services in rural India, behavioural and biological assessment relating to HIV at a private hospital-setting, and implications for future prevention and care programmes.

### Development of VCT services

We developed a VCT facility and HIV-education programme in a community that was, until our project began, naïve to similar services. This involved commitment to HIV prevention and care by hospital management, coordination of services within the hospital, dedication of physical space and other logistics to the VCT clinic, commitment of time of concerned personnel to training for the study, financial resources, and, most of all, commitment to the need for prevention and care services in the area. Furthermore, an existing health-information database helped identify areas to focus prevention programmes and monitor and follow up residents. We acknowledge that even one, let alone all, of these factors working together is rare in a rural area in India. Our efforts, such as those to create awareness about HIV prevention and value of VCT and individualized post-test counselling, were intensive in both human and financial resources, and current project funding was able to facilitate that. Furthermore, the flexibility of project and hospital staff to take on multiple tasks suggests that, in resource-limited settings, maximizing the potential of committed individuals is an important strategy to achieve public-health goals (18). We submit that, with planned allocation of available resources, efficiency in managing VCT services (27), and addressing client needs by bringing services closer to their location are important steps to bring benefit to a large population of under-served individuals. The participation of over 5,000 individuals in the VCT programme suggests this potential benefit and the potential strengths of the private sector in delivering these services. Further, the commitment from the Government to focus on rural areas for HIV prevention (23) underscores the need for similar future endeavours.

The second finding of this study is the lessons concerning behavioural and biological assessment in a rural population. The high rates of attendance at VCT, but low rates (1%) of return for test-seeking and low rates (18%) of responses to questions on reasons for test-seeking, are noteworthy and deserve discussion. We recruited individuals who visited a hospital with whom they have a long relationship. Some reasons for low rates of return could be this very relationship—individuals may have expected follow-up reminders and/or personalized attention as with other health conditions. A way of assessing this would have been to offer reminders and then assess return rates for collection of test-result. We did not do this to reduce the time between test-result and post-test counselling, and it is a limitation of the present study. Other studies, such as those in urban blood banks, also reported low rates of return (28). Understanding the reasons for low rate of return is an important gap to be filled. Observations of counselling practices to provide feedback or spot-testing for HIV that will eliminate long waits are examples of some steps that prevention programmers can take to understand and address low rates of return.

Low rates of responses to questions of reasons for test-seeking may also have resulted due to a combination of limited exposure to HIV-prevention information and an element of social desirability bias. Despite efforts in the community to disseminate information, our approaches to informing were limited to mass and/or large group message delivery. While these are popular, other individualized approaches, such as peer-led efforts (29) and individualized information sessions (15), have shown to increase participation in VCT efforts. Another reason for inadequate reports for reasons for test-seeking may be social desirability bias. Despite training in ethical principles and adherence to informed consent processes in this study, it is important to recognize that individuals may have participated in the study as it was being conducted in a familiar hospital. Our ethnographic data suggest that individuals expect to be informed about the testing processes and desire non-judgmental treatment of individuals seeking test. While we had trained counsellors who provide individualized counselling and sought to minimize the stigma and offered follow-up services to those who tested positive, we acknowledge that the potential of this bias is real in the study area. Studies have reported on the role of social desirability bias in the validity of responses (30), but only a few studies have been conducted in India. Research on the influence of the role of individuals in higher authority, such as doctors on behaviours of patients and on the perceived and real inducements offered to study participants (30), may highlight ways to improved validity of responses and to better understand participation in future studies. Understanding how participants think about research and the benefits of their participation in studies and, particularly, perceptions about the HIV-testing process can further add value to the literature from rural India.

The third finding involves implications to future HIV-prevention and care programmes in rural India. Our findings on reasons for test-seeking suggest that primary reasons cited for seeking an HIV-test were based on past sexual behaviours or because the participant felt sick. More men and those with risky behaviours, such as multiple partners before and after marriage, having sex with a sex worker or casual partner, and those who report alcohol-use before sex sought test because of their past sexual behaviour. Results of analysis of seeking test because they were sick also showed that more men than women sought care. Studies from urban Indian populations have reported that men reported high-risk behaviours, such as pre- and extramarital sex (31) and using alcohol more often than women (32). In the present study, 22% of men reported alcohol-use before sex. While we are limited by low response rates in this study, these findings suggest the prevalence of risk behaviours and signal the need for large scale, individual-focused programmes to bring the needed HIV-prevention and care services to this population. These efforts might focus on men and begin by understanding the contextual factors that facilitate risk behaviours. Some of these factors, such as knowledge of condom-use and ability to access condoms (33) when needed, need to be understood better from the male perspective. The low use of condom observed in this study is worrisome considering the epidemic in India and, particularly, in Andhra Pradesh state. This suggests that the current media messages and campaigns to promote condom-use might need to expand on the ‘Use condoms’ (34) or ‘Be safe’ messages (35). Barriers—individual and structural—to condom-use need to be better understood, and strategies to motivating individuals to use condoms despite these barriers that are being employed elsewhere in India (36) might be applicable to this and similar rural populations in India. The role of structural factors is also highlighted in the bivariate analysis of this study where we observed that those with higher income and education cited past behaviours and being sick as reasons for care-seeking. Poor access to financial and informational resources is a tangible concern among residents and health workers in rural area. Interventions might consider addressing these barriers as a way to promote sexual health.

Based on our findings, we conclude that VCT services and HIV-prevention education need to be designed considering gender differences in risk and healthcare-seeking. Additionally, private-sector practitioners should be aware of these differences and trained adequately to offer high-quality services. Their large presence in rural India demonstrates a willingness to work in impoverished settings where services are most needed. This asset needs to be better used.

In conclusion, we acknowledge some limitations of our study conducted in the context of care provision. As this was a sample of volunteers, there is a possibility of selection bias which might have drawn individuals either at higher risk or who had prior positive experiences seeking care at the Mediciti Hospitals. We also recognize that large sample sizes, as one in this study, can detect even small differences in outcomes. We limited our analysis to outcomes where this was not an issue to discuss areas of public-health significance and not simply statistical significance. Given that the participants had never been exposed to HIV-prevention efforts and that interviews were administered by a hospital counsellor, we may have elicited socially-desirable responses. We also acknowledge the role of secular trends in interpreting the data and in generali-zing these to the present day. Finally, we did not seek reasons for not returning for HIV-testing nor could we gather data from those who did not opt for VCT. We could not do this due to constraints of funding and time of counsellors and participants; however, we acknowledge that this provides valuable information to publicize VCT programme design in rural India.

## ACKNOWLEDGEMENTS

The study was funded by the World AIDS Foundation. The authors acknowledge the support of Mr. Purushottam who oversaw data management in the project.
